# Femtosecond Laser Fabrication of Curved Plasma Channels with Low Surface Roughness and High Circularity for Multistage Laser-Wakefield Accelerators

**DOI:** 10.3390/ma16083278

**Published:** 2023-04-21

**Authors:** Hongyang Deng, Ziyang Zhang, Min Chen, Jianlong Li, Qiang Cao, Xuejiao Hu

**Affiliations:** 1 The Institute of Technological Sciences, Wuhan University, Wuhan 430072, China; 2 Key Laboratory for Laser Plasmas (MOE), School of Physics and Astronomy, Shanghai Jiao Tong University, Shanghai 200240, China

**Keywords:** femtosecond laser, plasma channels, response surface method, sapphire, laser-wakefield accelerator

## Abstract

A multistage laser-wakefield accelerator with curved plasma channels was proposed to accelerate electrons to TeV energy levels. In this condition, the capillary is discharged to produce plasma channels. The channels will be used as waveguides to guide intense lasers to drive wakefields inside the channel. In this work, a curved plasma channel with low surface roughness and high circularity was fabricated by a femtosecond laser ablation method based on response surface methodology. The details of the fabrication and performance of the channel are introduced here. Experiments show that such a channel can be successfully used to guide lasers, and electrons with an energy of 0.7 GeV were achieved.

## 1. Introduction

The laser-wakefield accelerator has brought about a new era of highly stable quasi-monoenergetic electron beams that possess high quality [1,2,3]. These beams have wide applications, including desktop X-ray radiation, pumped detection, and femtosecond X-ray diffraction [4,5,6]. The existing accelerator technologies and radiation sources are associated with high costs. However, the introduction of laser plasma accelerators can greatly reduce these costs and show potential applications in the production of higher energies and higher-quality electrons, which could surpass the limits of traditional accelerators. The laser-wakefield accelerator utilizes an electron plasma wakefield with a large amplitude that is excited by an ultra-high-power laser in a gas plasma to accelerate charged particles. This accelerator can achieve up to 1000 times higher electron acceleration gradients compared to traditional high-frequency cavity accelerators, facilitating the development of ultra-compact desktop accelerators [7]. A laser-wakefield accelerator can achieve up to 7.8 GeV acceleration of electrons [8]. However, cascaded acceleration is required to obtain electron beams of TeV or higher energy [9]. Currently, the existing cascade acceleration schemes have complex acceleration structures, leading to difficulties in transferring electrons between accelerator stages, so the electron capture efficiency of the post-accelerator is low. Alternatively, the laser-insertion cascade scheme can be used, as it shortens the distance between the stage accelerators and eliminates the need for additional plasma lenses to converge the electron beam and plasma mirrors so as to reflect the laser [10]. This scheme reduces the scale of cascade acceleration and simplifies the acceleration process while improving the electron beam capture rate. In this scheme, the front end of the next channel is designed as a gradually curved geometric structure for guiding the laser, and the laser waist oscillation when entering the straight line is reduced to improve the electron beam capture rate, simplifying the acceleration process [11]. The laser-wakefield accelerator is pushing the boundaries of accelerator technology and showing great promise in revolutionizing hard X-ray free electron lasers and future TeV high-energy colliders.

An accelerator scheme requires precision-guided manufacturing of capillaries to improve the quality of capillary channels and extend their lifetime. To achieve this requirement, sapphire can be used as a substrate material owing to its high UV to mid-infrared transmittance and stable physicochemical qualities. The crystal structure of sapphire is due to the hexagonal lattice formed by both Al^3+^ and O^2−^ ions. However, the force of ionic bonding within the lattice bonding energy is dominated by the electrostatic interaction. As such, the C plane sapphire possesses the lowest bonding energy and the highest removal rate; this was derived from the distance between atomic layers of different crystalline planes of sapphire (C plane > M plane > A plane > R plane) [12,13]. The C plane has the highest fracture toughness value of 6.043 MPa m^1/2^, which indicates its superior performance in preventing crack initiation and propagation [14]. Consequently, the C plane sapphire was selected as the processing material for the accelerator scheme in this study, and monocrystalline sapphire having dimensions of 30 × 5 × 2 mm^3^ was considered a specimen (length × width × height). Moreover, the machined surface size was 30 × 5 mm^2^.

The capillary channel, which is a vital component in laser-wakefield acceleration, requires a minuscule inner diameter, a circular and uniform cross-section, and a smooth surface [15]. Sapphire is renowned for its exceptional hardness, thermal stability, and stable chemical composition and presents a unique challenge to traditional mechanical processing techniques. This can be resolved by implementing femtosecond laser ablation technology that can generate micro nanostructures in hard materials [16,17]. The femtosecond laser fabrication of a wakefield accelerator has proven to be a viable and highly promising solution [18].

Multiphoton or tunnel ionization is induced when the femtosecond laser impacts sapphire crystals, exciting electrons from the valence band to the conduction band. In the conduction band, avalanche ionization occurs when electrons absorb photon energy and collide with valence electrons [19]. Femtosecond laser pulses are ideal for high-resolution processing of hard materials since they have minimal heat transfer to the lattice and almost no thermal effects to consider during energy transfer. Processing such materials necessitates the use of a laser with a high-energy density that lowers processing accuracy and surface roughness; this fails to satisfy the criteria for optical devices. This limitation can be addressed using a three-wavelength, dual-position vibrating mirror scanning femtosecond laser micro-nanomachining system. To optimize the processing parameters, a univariate analysis of factors was carried out, followed by the construction of a second-order regression model using the response surface method [20]. The wet etching technique was used as a processing aid to identify optimal parameter combinations and auxiliary processes [21]. Finally, a dependable processing guideline was established to manage capillary channel processing.

## 2. Experiments

**Figure 1:**
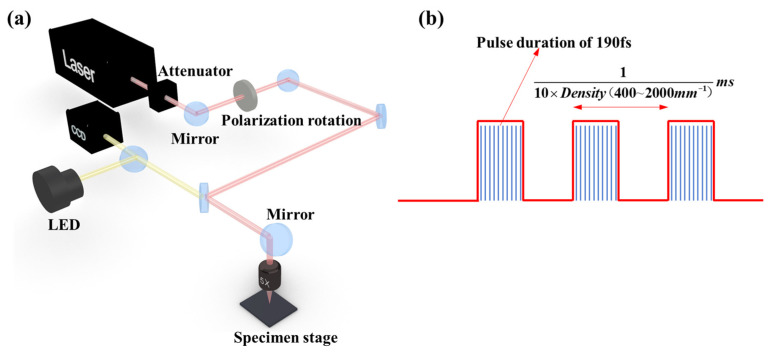
(**a**) Schematic diagram of the laser fabrication system and (**b**) burst-mode pulses.

The experimental setup employed to fabricate our product is captured in Figure 1a. The femtosecond laser micromachining system featured a Yb:KGW laser with a wavelength of 1026 nm, replete with a repetition frequency of 200 kHz and a full width at half maximum laser pulse of 190 fs. These parameters are determined to be the most appropriate based on a previous study [22]. A laser spot with a diameter of approximately 10-µm was generated using a 5× microscope featuring a numerical aperture of 0.14. Our sample was placed on a multi-axis precision positioning platform (American Aerotech Company, Pittsburgh, PA, USA), capable of nano-level precision, high resolution, and high repeatability. A charge-coupled device system was used to monitor the machining process. For greater insights, a white light interference 3D surface contour meter was used as our means of observation.

**Figure 2:**
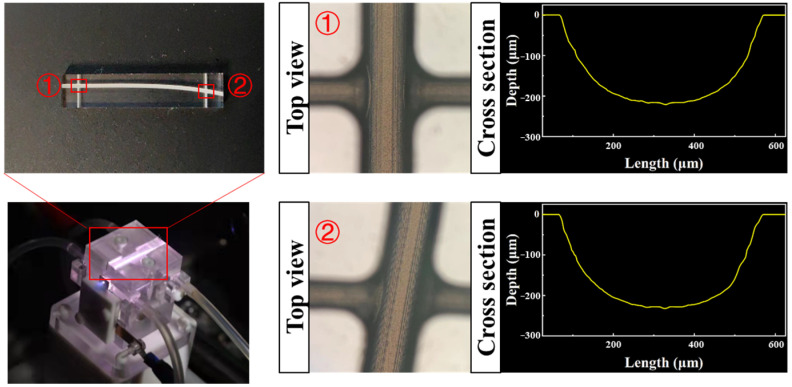
Curved plasma channels and the appearance and cross-section shapes of irradiation area.

Figure 1b illustrates the adopted burst-mode pulses. Each burst consisted of several sub-pulses with a consistent pulse duration of 190 fs and an adjustable repetition rate. Here, density is the number of machining points per millimeter. As shown in Figure 2, highly nuanced curved plasma channels with low surface roughness and high circularity were obtained.

**Figure 3:**
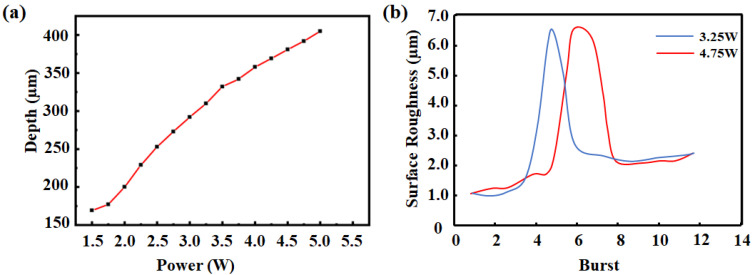
(**a**) The maximum channel depth as a function of laser power. (**b**) Surface roughness as a function of burst after hydrofluoric acid dissolves.

**Figure 4:**
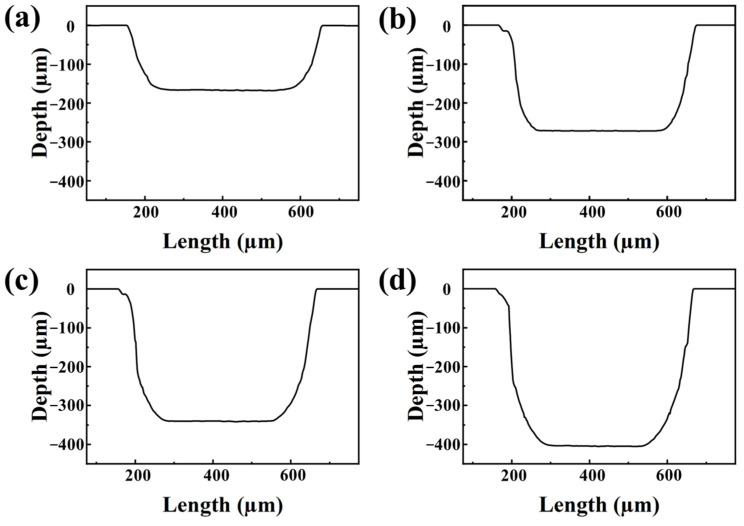
Cross-sectional morphology of channels processed with different laser powers (**a**) 1.75 W (**b**) 2.75 W (**c**) 3.75 W (**d**) 4.75 W.

The laser was investigated for channel creation. The femtosecond laser in sapphire material caused a surge of free electrons due to the avalanche ionization phenomenon. This resulted in increased free electron density, which ultimately caused the plasma frequency to be the same as the carrier frequency of the laser [23]. The creation of a deep channel requires a multi-layer processing method, and near-threshold laser power is ineffective. Thus, the effect of laser power on the processing of capillary channels was investigated. For this, the channel depth was gradually increased by increasing the laser power from 1.5 W to 5 W (the corresponding pulse intensity is 7.5 µJ–25 µJ) with a 0.25 W gradient. The variation of maximum channel depth with laser power is shown in Figure 3a. In addition, Figure 4 shows the deterioration of the edge shape measured using white light interferometric profilometry, but the bottom morphology of the channel remained unchanged. With respect to laser processing on metals, surface quality is affected mainly by the degree of overlap, i.e., the higher the degree of overlap, the lower the surface roughness, regardless of the material [24]. The above principles were considered when examining the mechanics of laser action on sapphire.

The inescapable consequence of the overlap rate on the processed surface is a matter of grave concern [24,25]. To understand the exact effect of certain parameters and avoid complications, the point density (number of processed points per millimeter length) is adjusted to control the overlap rate of the laser pulse. The point density is escalated at an unvarying scanning speed from 400/mm to 2000/mm, at an increment of 200 density, to gage the impact. The reduction in point density improved the quality of the morphology at the bottom and the edges of the capillary channel but decreased its depth. However, reducing the point density to an excessively low level caused an abysmal processing effect where the basic contour of the channel could not be replicated. Conversely, if the point density was set exceedingly high, it distorted the overall contour of the channel, and the removal rate was exceeded. Given that the point density is significant in determining the depth and roundness of the capillary channel, an optimum numerical interval of point density should exist. Considering the depth specifications of the channel, combining the point density and laser power dynamically results in the final process parameters.

Electronic excitation can be used to optically control the phase transition of solid materials. Excitation of approximately 10% of valence electrons results in the transition from a semiconductor to a metal [26]. The metal plasma state can be created in the focus of a typical narrow-focusing pulse. This pulse ionizes the focus volume almost instantaneously within one or more optical cycles by multiphoton absorption and emits a shock wave that quickly quenches, leading to a change in the chemical nature of different material phases.

When the laser is applied to sapphire, the crystal state of the surface is altered, and the degree of alteration is determined by the burst, which directly affects the quality of the capillary channels. Interestingly, the crystal state transformation of sapphire differs under varying pulse numbers of the femtosecond laser. When a single pulse femtosecond laser acts on sapphire at the current laser energy, the sapphire transforms from a single-crystal state to an amorphous state, and two uniform and amorphous regions emerge in the center of focus [27]. Consequently, the absorption capacity of sapphire becomes stronger due to the narrow band gap. Subsequent pulses furnish enough energy to precipitate polycrystalline sapphire [28,29,30]. Distinctly, multi-pulse femtosecond lasers may produce polycrystalline sapphire, which is more stable than the amorphous state and untraceable by hydrofluoric acid wet etching; the processing accuracy is wholly determined by femtosecond laser processing. However, the surface etching threshold of sapphire decreases significantly with the addition of ultrashort laser pulses, until it reaches an almost constant level owing to the incubation effect [31]. This indicates that a lower-power multi-pulse laser can be used to create plasma channels and improve the surface quality of channels. Burst is a critical factor in the processing of plasma channels. Therefore, we conducted numerous experiments involving several sets of comparisons to assess the explicit impact of burst on plasma channel quality. We set up two parallel experiments with different laser powers and employed a processing model comprising a cube of 300 × 300 × 100 µm^3^, where the surface roughness was gauged after treating the sample with 40% hydrofluoric acid for 1 h.

**Figure 5:**
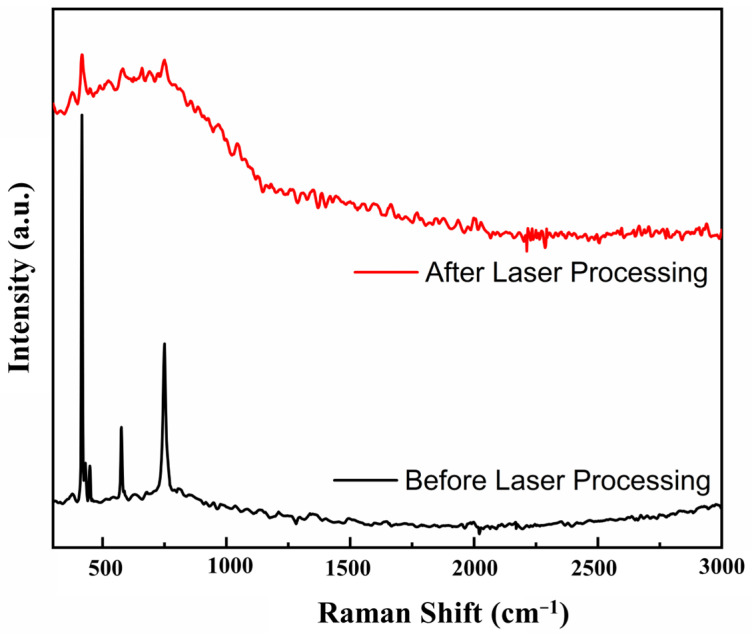
Raman Spectrogram of sapphire surfaces before and after laser processing.

The roughness of the sapphire undergoes a transformation under the influence of laser processing combined with wet etching. As shown in Figure 3b, the surface roughness of the sapphire initially increases and then decreases with different power outputs. Thus, sapphire remains essentially amorphous when the burst quantity is limited. In the procedure of the femtosecond laser equipment in our lab, the burst quantity parameter means how many laser pulses to output at once. However, as the burst quantity proliferates, sapphire density increases and roughness is augmented. In addition, at a certain critical burst quantity, amorphous sapphire absorbs sufficient energy to undergo polycrystalline precipitation. After processing, polycrystalline and amorphous coexist in a state of reality. When hydrofluoric acid dissolves nearly all the amorphous sapphire, the surface roughness undergoes a sudden spike compared to its previous state [32]. We measured the Raman spectra of the unprocessed sapphire sample, and the Raman Spectrogram obtained were consistent with those of single-crystal sapphire. Then the Raman spectra of the laser-processed sapphire were measured, and the comparison of the two is shown in Figure 5. It is obvious that the Raman spectrogram changed significantly. It could be determined that the crystalline phase of the sapphire had changed before and after processing. If the burst quantity exceeds a high enough threshold, sapphire exists only in its polycrystalline form, and surface roughness experiences an abrupt decline, reaching a constant value. This presents the accuracy achievable with femtosecond laser processing using current energy. We observed that the maximum roughness occurs at a relatively low burst quantity with high-power laser processing, aligning with theoretical predictions. As the single pulse energy increases, the amorphous to polycrystalline transition becomes more effortless, and the roughness transition point drifts forward. Indeed, the experiment results in Figure 3b conducted that diminishing the burst quantity significantly augments channel surface quality compared to lowering the laser power output.

Response surface methodology was employed to analyze the interactions between factors due to the correlation between them. The experimental design adopted a central composite including three factors: laser power, burst number, and point density; sapphire microchannel quality was considered the response value. Central composite design (CCD) is the most popular second-order design used by researchers, and the estimation of the optimum value is combined with CCD by the response surface method. The number of experimental runs of CCD is calculated by the following formula:

(1) $$N=2^{K}+2K+C_{p}$$

This experiment includes three variables. For these variables (*K* = 3) and two levels (− and +), the total number of 20 trials is given by the Formula (1): 8 factor points, 6 axial points, and 6 central points. Therefore, it included 20 runs. We quantify the mass by using Equation (2):

(2) $$Q=((1-\frac{\left|Depth\right|}{250})+Roundness+(1-\frac{Roughness}{Roughness_{\max}}))\times \frac{100}{3}$$

## 3. Results and Discussion

### 3.1. Regression Model of Plasma Channel Mass

The response surface experiment data was statistically analyzed using the Design Expert software. The software comprises different models such as linear, interactive, quadratic, and cubic, and we chose the quadratic model in this study to develop a regression model of laser power, burst number, and point density. With *P*, *B*, and *D* representing laser power, burst number, and point density, respectively, the regression model of channel mass is developed as:

(3) $$\begin{array}{c} \;\;Q=48.63+15.8P-7.1D-8.2B-5.87PD-12.62PB\;+4.13DB-14.32P^{2}+1.18D^{2}-0.3182B^{2} \end{array}$$

### 3.2. Feasibility of Fitted Model

As presented in Table 1, the *F* test values of the second-order CCD regression model were studied to ascertain the feasibility of the fitted model. The *F*-value increased in tandem with a decrease in the *p*-value, making the selected second-order quadratic model terms more significant. A *p*-value lower than the 0.05 threshold translated to significant regression with a whopping 95% confidence level. Anything greater than a 0.1000 *p*-value was insubstantial for all regression models. As depicted in Table 1, the regression model proved to be distinctly significant with a *p*-value of 0.0003 at the noteworthy level. The lack of fit was relatively unimportant, given a *p*-value greater than 0.05 at the noteworthy level.

**Figure 6:**
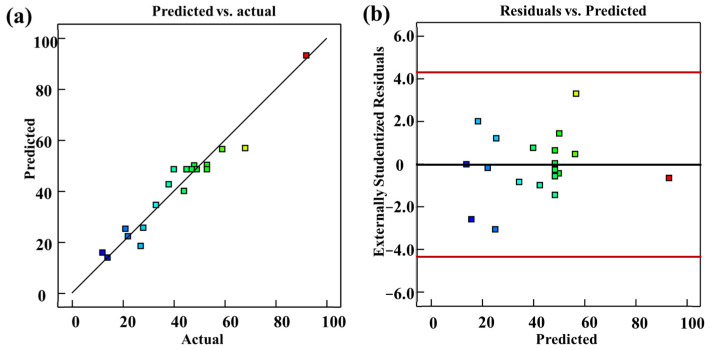
(**a**) Predicted probability vs. actual probability of quadratic regression model; (**b**) the residuals of quadratic regression model.

A probability graph was plotted as the hallmark of the model’s importance for a more insightful and revealing perspective. Figure 6a presents a visual representation of responses garnered from 20 experiments using CCD. Noticeably, these points are close to the diagonal line, underscoring the feasibility and workability of the developed model. Moreover, the residual plot shown in Figure 6b reinforces the salient points gleaned from Figure 6a. In essence, the residuals are evenly distributed around the zero line, with an insignificant gap between the actual data and the predicted data.

In the evaluation of the accuracy of the model, the correlation coefficient R^2^ becomes an essential factor after the response surface optimization and equation coefficient calculation via the least squares method. A higher value of R^2^ signifies better accuracy for the model. In this study, we have established a degree of agreement between the predicted values and experimental values of the polynomial model. The correlation coefficient, R^2^, for the model is 91.73%, which excellently explains the experimental accuracy of greater than 75%. Moreover, the adjusted R^2^ value for the model is 84.29%, and the difference between the adjusted and actual R^2^ values remains within 0.2. Therefore, the precision was found to exceed 4, signifying a strong signal, and no other potential factors affected the model. This proves the validity of the signal from the model. These results demonstrate a significant correlation between the experimental and predicted data generated by the model. The regression model comprising laser power, burst number, and point density established in this section is profoundly consistent with the experimental results.

### 3.3. Parameter Optimization

**Figure 7:**
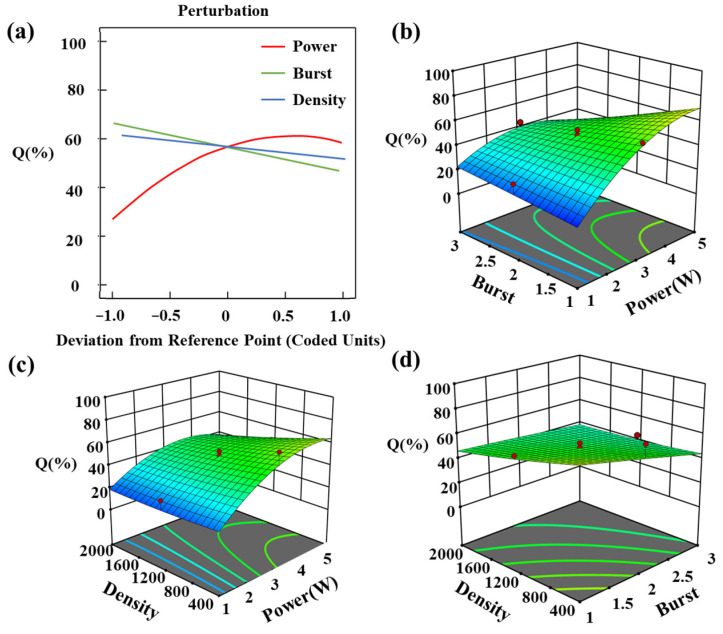
(**a**) Effect of a single process parameter on channel quality. The interaction between process parameters and channel quality is influenced by (**b**) burst and power, (**c**) density and power, and (**d**) density and burst.

Figure 7a describes the effect of single-factor variables on channel quality. Within a certain range of bias, the channel mass increases and then decreases with the increase in laser power, while the channel mass decreases with the increase in burst number and density. The regression model of channel mass is related to the interaction between process parameters as the equation also contains non-linear terms.

Figure 7b–d shows the interaction effect of process parameters on channel quality, which is consistent with the effect of single parameters on the response. We optimized the process parameters for femtosecond laser processing of sapphire plasma channels using the established regression model. The optimization target was set to mass maximization, combined with the specific experimental results for finetuning. The optimization results for the process parameters were found to be 4.75 W power, 600 density, and single burst.

### 3.4. Product Inspection

We used a femtosecond laser with optimal parameters to fabricate a sapphire microchannel. The fabricated channel is well shaped, has a depth of 250 µm, and has good cross-sectional roundness and smooth edges. The position with the highest surface roughness is the junction of the channel, but we have high requirements for the roughness at the junction. To detect the quality of the inner wall surface of the channel, we selected four 100 × 100 μm^2^ areas at both junctions of the channel and measured the surface roughness. Our findings showed that the Sa (surface arithmetic average height) was lower than 0.5 μm; it is derived from computer measurements.

We conducted tests using a capillary-based laser-wakefield accelerator at the Laser Plasma Laboratory of Shanghai Jiao Tong University to ascertain whether the plasma channel could withstand practical applications. A laser-matched plasma density distribution prevents the laser from defocusing in the capillaries [33]. When the discharge voltage is between 18 kV and the back pressure is between 10 psig, a plasma channel with a transversely parabolic profile can be generated. Low-intensity laser guidance was achieved under perfect guidance conditions [34,35]. Our assessment revealed that a low-intensity laser safely exits the capillary with a circular wave plane distribution that mirrored its entry into the capillary. This confirmed that the channel was a perfect match for the laser. Moreover, we also tested the guiding ability of the channel for a high-intensity laser (with an energy of 3 J). Experiments show that a high-quality energetic electron beam with a maximum energy of 0.7 GeV can be observed when the laser is well guided in the channel [35]. This indicated that the laser experienced no significant deformation or diffusion within the capillary channel.

## 4. Conclusions

Our study shows that the power of the laser holds great sway over the very depths of the channel, while the number of bursts holds an equally significant influence over the surface roughness of the channel. In addition, the density of processing points affects the roundness of the channel. The response surface method was used to analyze and obtain the optimal processing parameters of the sapphire capillary because of interactions between the parameters. Finally, the sapphire channels with a depth of 250 µm, a surface roughness below 0.5 µm, and a roundness within 5 µm were successfully developed using the laser-wakefield accelerator device. A plasma channel was indicated to be successfully formed in the capillary at high-voltage discharge (18 kV), and 0.7 GeV energetic electron beam acceleration in such a curved plasma channel was successfully observed.

## Figures and Tables

**Table 1:** *F* test results for CCD for all responses.

Source	df	*F*-Value	*p*-Value	Remarks
Model	9	12.32	0.0003	Significant
A-P	1	43.84	<0.0001	
B-B	1	11.81	0.0064	
C-D	1	8.85	0.0139	
AB	1	22.39	0.0008	
AC	1	4.85	0.0523	
BC	1	2.39	0.1531	
A^2^	1	9.90	0.0104	
B^2^	1	0.0049	0.9456	
C^2^	1	0.0675	0.8004	
Lack of Fit	5	4.96	0.0517	Not significant

## Data Availability

Data available on request due to restrictions eg privacy or ethical. The data presented in this study are available on request from the corresponding author. The data are not publicly available due to they contain proprietary information or trade secrets.
